# Increased risk for aseptic meningitis after amoxicillin or amoxicillin‐clavulanic acid in males: A signal revealed by subset disproportionality analysis within a global database of suspected adverse drug reactions

**DOI:** 10.1002/pds.4707

**Published:** 2018-12-17

**Authors:** Rebecca E. Chandler

**Affiliations:** ^1^ Research Section Uppsala Monitoring Centre Uppsala Sweden

**Keywords:** amoxicillin, aseptic meningitis, disproportionality analysis, gender, pharmacoepidemiology, signal detection, subgroups

## Abstract

**Purpose:**

Drug‐induced aseptic meningitis (DIAM) is an inflammation of the membranes of the central nervous system caused by certain medications. It is a rare clinical entity whose risk factors are not yet fully elucidated. A local pattern of disproportionality within a global database of suspected adverse drug reactions (ADRs) revealed an increased reporting of aseptic meningitis and amoxicillin‐clavulanic acid (AC) in males. The aim of this report is to explore the clinical probability of a higher risk in males to support the use of statistical methods to identify subgroups at risk for adverse drug reactions.

**Methods:**

Disproportionality analysis was performed for all drug‐adverse event (AE) pairs in the entire database and for the subsets of males and females. AC‐aseptic meningitis was highlighted for an increased disproportionality in the male subgroup in the absence of an elevated disproportionality measure for the database overall. A clinical review was undertaken.

**Results:**

Clinical review revealed a similar statistical pattern of gender difference observed for amoxicillin, evidence to suggest a delayed type 4 hypersensitivity reaction with Th1 cells as a mechanism for amoxicillin‐aseptic meningitis, the existence of sex differences in immune responses (Th1/Th2), and an analogous increased risk of drug‐induced liver injury by AC in males.

**Conclusions:**

Subgroup disproportionality analysis has revealed a larger than expected number of reports of aseptic meningitis after amoxicillin and AC in males. Evidence synthesis supports the statistical finding. Further exploration of spontaneous databases with more extensive analyses could usher in a new era of “precision pharmacovigilance.”

**KEY POINTS**

- A higher than expected number of reports of aseptic meningitis after amoxicillin and amoxicillin‐clavulanate have been received from males into a global database of suspected adverse drug reactions.
- Sex differences in immune responses, such as delayed type hypersensitivity reactions, which can be a mechanism for drug induced aseptic meningitis, have been described.
- More extensive analyses of databases of spontaneous adverse drug reaction (ADR) reports could be used to assist in the identification of potential risk factors for ADRs.

## 1 INTRODUCTION

Drug‐induced aseptic meningitis (DIAM) is an inflammation of the membranes of the central nervous system caused by the administration of certain medications. Clinically, patients experience symptoms of fever, headache, and changes in mental status. Diagnostically, analysis of cerebrospinal fluid (CSF) reveals the presence of an increased number of white blood cells, elevated protein level, but a normal glucose level. All microbiological cultures are negative. The diagnosis of DIAM is often one of exclusion of more common infectious causes of meningitis.

Given the rarity of its occurrence, the incidence of DIAM is unknown and risk factors for it have not been fully elucidated. However, cases of DIAM caused by amoxicillin and amoxicillin‐clavulanic acid (AC) have been extensively reported in the literature, and a series of reviews have summarised a number of aspects of DIAM from the published case reports. The most commonly reported agents to cause DIAM are non‐steroidal anti‐inflammatory agents, antimicrobials, intravenous immunoglobulins, OKT3 antibodies, and anticonvulsants. The time‐to‐onset report can vary between several minutes to several months. Systemic lupus erythematous is the most common underlying condition reported with DIAM. Additionally, recurrent episodes of DIAM have been described in multiple case reports; such patients are predominantly females and a large proportion have underlying inflammatory diseases.^1^, ^2^

## 2 METHODS

Disproportionality analysis was performed in VigiBase, the global database for individual case safety reports, for all drug‐adverse event (AE) pairs in the entire database and for the subsets of males and females using methodology for local pattern discovery.^3^ AC‐aseptic meningitis was highlighted for an increased disproportionality in the male subgroup in the absence of an elevated disproportionality measure for the database overall. IC_0005_ is the lower endpoint of a 99.9% credibility interval for the information component (IC), and as such was used to support analysis of subgroup specific associations between substances and AE. Initial manual assessment expanded the review to include amoxicillin, which included both an increased disproportionality in males as well as in the entire database. A thorough clinical review of the drug‐ADR‐risk group triplet was undertaken, which included further review of spontaneously reported ADRs, the reported case series, as well as the medical literature for evidence of biological plausibility of an increased risk in males.

## 3 RESULTS

As of September 9, 2018, there was a total of 2695 individual case safety reports in VigiBase, which included the preferred term (PT) “meningitis aseptic”; 56.7% of the reports described females, 37.3% males, and in 6.0% of the reports, gender was not stated. The most common drugs reported in association with aseptic meningitis were human immunoglobulins (35.3%) and ibuprofen (6.6%). There was a total of 47 reports with amoxicillin (26 males, 21 females) and 24 for AC (18 males, 6 females).

For the combination of amoxicillin and the MedDRA PT, meningitis aseptic, 26 cases were observed compared with seven expected in males (IC_0005_ 0.80), and 21 cases were observed and nine expected in females (IC_0005_–0.14). Further investigation revealed a similar pattern of gender difference for AC; 18 cases were observed compared with seven cases expected in males (IC_0005_ 0.04); six cases were observed and nine expected in females (IC_0005_–3.14).

After removing suspected duplicate reports, there were a total of 36 unique cases reported in males (Table 1). Ages ranged from 1 month to 86 years. Reports were received from many countries, including the United States, France, Germany, Belgium, Switzerland, Czech Republic, Italy, Spain, Portugal, Japan, and New Zealand. The case series is remarkable for the large proportion of reports that offer information on indication, time‐to‐onset, dechallenge/rechallenge, as well as narrative information on clinical course. The reported indications for use of amoxicillin and/or AC were varied and according to licensed indication/treatment guidelines; there was no suggestion of a difference in use of the antibiotics between reports from males and females.

**Table 1:** Case reports of amoxicillin/clavulanic acid and aseptic meningitis in males in VigiBase

Case	Age/sex	Medications	Reactions	Indication	Dose	Time to onset	Notes/outcome
1	25 y/M	Amox/Clav (S)	Aseptic meningitis Headache	Furuncle	1 gm, 3 per d, oral	13 d	CT/MRI/EEG within normal limits. LP (Lyme, herpes) negative. Ruled out limbic encephalitis.
2	77 y/M	Amox/Clav (S) Acetylsalicyclic acid (C) Simvastatin (C) Pantoprazole (C)	Aseptic meningitis	Bursitis	2220 mg, 3 per 1 d, oral	3 d	MRI/EEG/angiogram LP (HIV, Borrelia, syphilis, herpes, enterovirus, tick borne virus, varicella virus, parvovirus, flavivirus) negative Positive dechallenge.
3	63 y/M	Amox/Clav (S) Amlodipine; valsartan (C) Hydrochlorothiazide (C)	Aseptic meningitis Cephalgia	Bronchitis	1 dosage form, 2 per d, oral		LP consistent with aseptic meningitis. Multiple serologies for infectious and autoimmune diseases negative. Positive dechallenge. Positive rechallenge (one prior episode)
4	79 y/M	Amox/Clav (S)	Aseptic meningitis	Stomatitis			Present and past extensive evaluations for infectious and autoimmune diseases negative. Positive rechallenge (two prior episodes). Published case report. Militao et al.^6^
5	1 m/M	Amox/Clav (S)	Aseptic meningitis Feeding disorder neonatal Epilepsy Erythrocytes decreased Fever Haemtocrit decreased Haemoglobin decreased Leukocyte count decreased	Infection prophylaxis	2.5 ml, 2 per d	2 d	Had been treated for E.coli sepsis for 3 wk prior. Discharged on amox/clav as outpatient. Returned to hospital after third oral dose with meningitis.
6	58 y/M	Amox/Clav (S)	Aseptic meningitis	Skin infection		1 d	LP negative. Positive dechallenge. History of two previous rechallenges. Published case report: Preito‐Gonzalez et al.^7^
7	31 y/M	Amox/Clav (S)	Meningitis aseptic Headache Cervical pain Consciousness abnormal	Otitis media acute	1000 mg, 3 per d	11 d	
8	66 y/M	Amoxicillin (S) Amox/Clav (S)	Meningitis aseptic	Dental pain		4 d	CT negative. LP with lymphocytic pleocytosis. History of two previous episodes. Reported by allergist. Allergy skin tests with beta lactam antibiotics negative. Published case report. Alarcon et al.^8^
9	63 y/M	Amox/Clav (S)	Meningitis aseptic				
10	35 y/M	Amox/Clav (S) Ibuprofen (S)	Meningitis aseptic	Acute tonsillitis	‐, 3 per 24 h 600 mg, 3 per 24 h	5 d	Positive dechallenge LP with lymphocytic predominance
11	72 y/M	Amox/Clav (S) Terazosin (C) Moxifloxacin (S) Ceftriaxone (S) Aciclovir (S) Ampicillin (S)	Lymphocytic meningitis Anorexia Constipation Headache Consciousness decreased Vomiting projectile Shivering Temperature elevation Neck stiffness	Pyrexia with respiratory symptoms	875 mg, 3 per 1 d	8 d	Medical history of polyarthritis rheumatica, oesophagitis, colorectal polyp, and prostatism. All other antimicrobials added after patient hospitalised, although listed as “suspected.” Positive dechallenge and rechallenge
12	82 y/M	Amox/Clav (S) Terbutaline (S) Heparin (S) Macrogol 4000 (S) Ipratropium (S) Ceftriaxone (S) Spiramycin (S) Influenza vaccine (C)	Lymphocytic meningitis	Bronchopneumonia	Oral	8 d	Medical history of hypertension, cardiac insufficiency, diabetes, history of bladder cancer, and nephrectomy. Other medications in narrative: Simvastatin, molsidomine, diltiazem, irbesartan, clopidogrel, sitaglipine, fluticasone, and salmerterol. Hospitalised and treated for pneumonia with ceftriaxone and spiramycin for 4–5 days. Discharged on Augmentin. Returned to hospital with fever and confusion. LP and EEG performed. Positive dechallenge.
13	65 y/M	Amox/Clav (S) Amoxicillin (S) Pneumococcal vaccine (S) Influenza vaccine (S) Candesartan (C) Moxonidine (C) Pantoprazole (C) Oxybutynin (C) Methionine (C)	Meningitis aseptic Injection site inflammation	Injection site inflammation	**‐,‐** oral 2 gm, 6 per d IV	4–5 days	Medical history: Paraplegia, type 2 diabetes. Injection site reaction after vaccination, treated with amox/clav. LP with lymphocytes and elevated protein. Cultures and pcrs negative.
14	86 y/M	Amox/Clav (S) Amoxicillin (S) Celiprolol (C)	Meningitis aseptic	Complication of internal prosthetic device, implant, and graft	2 gm, − per d PO 12 gm, 1 per d IV		Recovered
15	/M	Amoxicillin (S)	Meningitis aseptic	Cellulitis	Oral	2 d	Positive dechallenge
16	74 y/M	Amoxicillin (S) Ibuprofen (S) Codeine (S) Caffeine; Papaver somniferum; paracetamol (S)	Aseptic meningitis	Prophylactic	Oral	7 d	Recovered with withdrawal of all meds. “Negative rechallenge with paracetamol makes it possible to exclude it from suspect treatments”
17	62 y/M	Amoxicillin (S)	Aseptic meningitis	Antibiotic prophylaxis	Oral	6 h	Positive dechallenge. Positive rechallenge (one prior episode). Extensive evaluation. No evidence of type 1 or type 2 hypersensitivity. Published case report: Kastenbauer et al.^5^
18	75 y/M	Amoxicillin (S)	Tonic–clonic epilepsy Lymphocytic meningitis	Tooth infection	Oral	2 d	Positive dechallenge. Positive rechallenge (two prior episodes).
19	65 y/M	Amoxicillin (S) Amox/Clav (S) Tiaprofenic acid (S) Hydroxyzine (C)	Meningitis aseptic	Tooth pain Tooth abscess	Oral	5 d	Extensive evaluations of CSF, including entervirus, herpes, EBV, CMV, Lyme, and syphilis. Positive dechallenge.
20	52 y/M	Amoxicillin (S)	Aseptic meningitis	Bronchitis	Oral	11 d	Admitted with confusion, afebrile. EEG and LP performed. Initially treated with IV amoxicillin and acyclovir. Positive dechallenge.
21	78 y/M	Amoxicillin (S)	Confusional state Malaise Consciousness loss Aseptic meningitis	Pneumopathy	3 g, 1 per 1 d. Oral	1 d	LP performed. PCRs negative. High cells and protein. Positive dechallenge.
22	20 y/M	Amoxicillin (S) Codeine; paracetamol (C)	Aseptic meningitis	Dental disorder prophylaxis	2 g, 1 per Oral	2 d	Elevated IgE to ampicillin. Positive dechallenge. Positive rechallenge (three prior episodes with penicillin agents)
23	46 y/M	Amoxicillin (S)	Aseptic meningitis	Suspicion of Lyme disease	Oral	1 day	Positive dechallenge.
24	72 y/M	Amoxicillin (S)	Meningitis aseptic Upper respiratory tract infection	Oropharyngeal pain	500 mg tid Oral		Positive dechallenge.
25	42 y/M	Amoxicillin (S) Paraaminobenzoic acid (C) Ibuprofen (C)	Meningitis aseptic	Acute upper respiratory infection, unspecified	1.5 g per 1 d	3 d	Positive dechallenge.
26	17 y/M	Amoxicillin (S) Ibuprofen (S)	Meningitis aseptic		Oral	8 d	No narrative
27	55 y/M	Amoxicillin (S)	Meningitis aseptic	Prophylaxis	500 mg Oral		Positive dechallenge. No narrative
28	55 y/M	Amoxicillin (S)	Meningitis aseptic Phonophobia Tachycardia				Positive dechallenge. No narrative
29	55 y/M	Amoxicillin (S)	Meningitis aseptic Headache Chills Pyrexia	Antibiotic prophylaxis	500 mg single dose		Positive dechallenge. No narrative
30	44 y/M	Amoxicillin (S) Amox/Clav (S)	Meningitis aseptic Gingival pain Influenza Lymphadenopathy No therapeutic response Rash maculo‐papular Sinusity	Tooth abscess Sinusitis	Oral		Amoxicillin duration 7 d, Amox/Clav duration 12 d. Positive dechallenge recorded for amox/clav No narrative
31	55 y/M	Amoxicillin (S)	Meningitis aseptic	Dental disorder prophylaxis	500 mg, 1 per 1 d Oral		Positive dechallenge No narrative
32	86 y/M	Amox/Clav (S) Hydrochlorothiazide;Irbesartan (S) Clopidogrel (C) Metoprolol (C) Amiodarone (C) Tolterodine (C) Metformin (C) Simvastatin (C) Levothyroxine (C) Insulin lispro (C)	Meningitis aseptic Confusion Vomiting Sodium depletion	Bronchitis/pneumonia	Oral	4 d	Positive dechallenge Positive rechallenge
33	20 y/M	Amoxicillin (S) Paracetamol (C)	Meningitis aseptic	Acute pharyngitis	2 g, − per d Oral	6 d	Positive dechallenge No narrative
34	80 y/M	Amox/Clav (S) Amoxicillin (S) Clarithromycin (C)	Meningitis aseptic Tooth ache Urinary tract infection Lyme disease				No narrative
35	72 y/M	Amoxicillin (S)	Meningitis aseptic Unevaluable event Photophobia				No narrative
36	60 y/M	Amoxicillin (S) Ibuprofen (S) Simvastatin (C) Amlodipine (C) Hydrochlorothiazide (C) Folic acid (C)	Meningitis aseptic			Oral	No narrative

Abbreviations: CMV, Cytomegalovirus; CT, computed tomography; CSF, cerebrospinal fluid; EBV, Epstein–Barr virus; EEG, electroencephalography, HIV, human immunodeficiency virus; IV, intravenous; LP, lumbar puncture; MRI, magnetic resonance imaging; PO, per os. The table includes a line listing of all reports included in the analysed case series. (S) and (C) in the Medications column designate if a drug was reported as Suspected or Concomitant in relation to the ADR. All Reactions and Indications are encoded in MedDRA terminology.

DIAM has long been considered to be caused by one of two mechanisms: (a) direct chemical irritation of the meninges by drugs directly administered into the CSF, or (b) immunological hypersensitivity reactions. All types of immunological hypersensitivity reactions (types 1‐4) have been implicated in various case reports of DIAM, suggesting that mechanisms may be different for the various types of drugs.^4^

In the case of amoxicillin and AC, there is growing evidence that it is a delayed type 4 hypersensitivity or T‐cell mediated hypersensitivity that underlies its associated cases of aseptic meningitis. Kastenbauer et al described a case of a 62‐year‐old man with aseptic meningitis after AC, in which additional investigation did not reveal evidence of an underlying type 1 or type 3 hypersensitivity mechanism.^5^ More recently, Castagna et al reported in vitro enzyme‐linked immunosorbent (ELISPOT) assay results in two women with amoxicillin DIAM, demonstrating drug‐specific cells producing high quantities of Interferon gamma (IFN)–gamma and gamma‐ray bursts (GrB). IFN‐gamma is a key Th1‐type cytokine released from activated T cells in drug‐induced hypersensitivity.^9^

There is growing recognition of the existence of sex differences in immune responses. Contributing factors to the differential development and function of the immune system between males and females include sex chromosome genes and sex hormones as well as nutritional status and composition of the microbiome in the gastrointestinal tract. The results of these differences in immune responses is evidence of the differential susceptibility of males and females to autoimmune diseases, malignancies and infectious diseases, and of responses to vaccination. More specifically, there is evidence that females have higher CD4+ T cells and greater T cell activation and proliferation while males have higher CD8+ T cell frequencies. Females tend to be polarised towards Th2‐type responses, while males are more Th1‐biased and have more regulatory T cells.^10^ With the previously presented evidence for Th1‐type response central to the type 4 hypersensitivity of amoxicillin DIAM, it therefore follows that men may be at greater risk than females, as suggested by the disproportionality statistics.

An analogy can be found with drug‐induced liver injury (AC‐DILI) and AC. deLemos et al examined a large cohort of AC‐DILI cases prospectively enrolled from the United States DILI Network. A total of 117 cases of AC‐DILI were identified and compared with the remaining 921 cases in the registry. 62% of DILI cases ascribed to AC occurred in males, compared with 39% of DILI ascribed to all other antimicrobials. Of 31 liver specimens available for pathologic examination, almost all revealed immune‐allergic features (21) with eosinophils, 28 with granulomas, thought to be caused by infiltration of portal triads within the liver by cytotoxic CD8+ cells.^11^ Review of the SmPC for AC reveals information regarding this risk group: “Hepatic events have been reported predominantly in males and elderly patients and may be associated with prolonged treatment” (Section 4.4 Special warnings and precautions for use).^12^

## 4 DISCUSSION

Disproportionality analyses within spontaneous reporting systems are typically employed to identify potential causal relationships between drugs and adverse drug reactions. Previous work has shown that disproportionality analyses within subsets may reveal local patterns not detected in routine statistical screening of large databases. Such an analysis within a global database of suspected adverse drug reactions has revealed a larger than expected number of reports of aseptic meningitis after AC and amoxicillin in males. Biological plausibility for the statistically detected signal is supported by data suggesting a T‐cell mediated mechanism for amoxicillin‐induced aseptic meningitis as well as evidence of sex differences in immune responses, specifically increased frequencies in males of type 4 hypersensitivity reactions of a Th1‐type and/or involving a predominance of CD8+ cells. Further elucidation of the interindividual variation in immune responses may lead to better understanding of drug hypersensitivity reactions.

Risk characterisation is typically performed at the time of clinical signal assessment, looking for any obvious patterns between the patients described within the case series. This exercise has revealed the potential for risk identification at the level of statistical screening within large databases. Further exploration of spontaneous databases with more extensive analyses could usher in a new era of “precision pharmacovigilance.”

## ETHICS STATEMENT

The authors state that no ethical approval was needed.

## CONFLICT OF INTEREST

The opinions expressed in this piece are not necessarily those of the national pharmacovigilance centres of the WHO Programme for International Drug Monitoring or of the WHO.

The author has no conflicts of interest that are directly relevant to the content of this piece.
